# MYEOV Is a Novel Marker of Differentiated Corneal Epithelium

**DOI:** 10.1167/iovs.66.14.7

**Published:** 2025-11-04

**Authors:** Yoshiko Fukuda, Chao Zhang, Bruce R. Ksander, Vickie Y. Jo, Christine G. Lian, George F. Murphy, Markus H. Frank, Natasha Y. Frank, Yuzuru Sasamoto

**Affiliations:** 1 Department of Ophthalmology, Chobanian & Avedisian School of Medicine, Boston University, Boston, Massachusetts, United States; 2 Department of Medicine, Chobanian & Avedisian School of Medicine, Boston University, Boston, Massachusetts, United States; 3 Massachusetts Eye and Ear Infirmary, Schepens Eye Research Institute, Boston, Massachusetts, United States; 4 Department of Pathology, Brigham and Women's Hospital, Boston, Massachusetts, United States; 5 Transplant Research Program, Boston Children's Hospital, Boston, Massachusetts, United States; 6 Harvard Stem Cell Institute, Harvard University, Cambridge, Massachusetts, United States; 7 Harvard Skin Disease Research Center, Department of Dermatology, Brigham and Women's Hospital, Boston, Massachusetts, United States; 8 School of Medical and Health Sciences, Edith Cowan University, Perth, Western Australia, Australia; 9 Division of Genetics, Brigham and Women's Hospital, Boston, Massachusetts, United States; 10 Department of Medicine, VA Boston Healthcare System, Boston, Massachusetts, United States; 11 Department of Ophthalmology, University of Washington, Seattle, Washington, United States

**Keywords:** corneal epithelium, MYEOV, KRT12, PAX6, KLF4

## Abstract

**Purpose:**

Myeloma overexpressed gene (MYEOV) was initially identified as a gene amplified in several malignancies and was found to promote cell proliferation and metastasis. Our previous comparative RNA sequencing and epigenetic analyses revealed high MYEOV levels in differentiated corneal epithelial cells and showed that TET2 epigenetically regulated MYEOV expression. In the current study, we aimed to further characterize the expression and regulation of MYEOV in the human ocular surface.

**Methods:**

MYEOV expression was examined by immunostaining of the human corneas and by analysis of the publicly available single-cell RNA sequencing data. Gene knockdown (KD) of *MYEOV* and the regulators of corneal epithelial differentiation, *PAX6* and *KLF4*, in in vitro–expanded corneal epithelial cells was performed using siRNA transfection. Protein expression levels were examined by western blot. *MYEOV* KD cells were subjected to colony-forming and EdU cell proliferation assays and RNA sequencing analysis.

**Results:**

Human cornea immunostaining revealed strong MYEOV expression in the KRT12-positive differentiated corneal epithelial cells, whereas the majority of KRT13-positive differentiated conjunctival epithelial cells were MYEOV negative. MYEOV expression was not detected in the other surface ectoderm-derived epithelia, such as skin and oral mucosa. Both *PAX6* KD and *KLF4* KD resulted in a reduction of MYEOV and KRT12 protein expression. *MYEOV* KD decreased cell proliferation.

**Conclusions:**

Our study revealed specific high MYEOV expression in KRT12-positive corneal epithelial cells among surface ectoderm-derived epithelia. Similar to KRT12, MYEOV expression is regulated by PAX6 and KLF4. Functionally, MYEOV regulates the proliferation of transient amplifying cells in the cornea.

The cornea is composed of three main layers: epithelium, stroma, and endothelium, and it is separated from the conjunctiva by a circular limbal region. Keratin 12 (KRT12) has been commonly used as a specific marker of differentiated corneal epithelium.^1^^–^^3^ Except for the ectopic corneal epithelial cells in the conjunctiva,^4^ KRT12 is not expressed in other surface ectoderm-derived epithelia, such as conjunctiva, epidermis, and oral mucosa.^2^^,^^5^^,^^6^ No other known protein with similar expression specificity has been described to date. For example, KRT3 is specific to the corneal epithelium of the eye but can also be found in the oral mucosa.^7^ Although other genes, such as ALDH3A1 and CLU, are highly expressed in corneal epithelium,^8^ they are also detected in other surface ectoderm-derived epithelia.^9^^–^^12^

Myeloma overexpressed gene (MYEOV) was initially reported as a gene associated with multiple myeloma.^13^^,^^14^ MYEOV was also identified as a gene amplified in several other malignancies, such as breast cancer, esophageal squamous cell carcinoma, pancreatic ductal adenocarcinoma, non-small cell lung cancer, and neuroblastoma,^15^^–^^19^ where it promotes cell proliferation and metastasis.^17^^–^^19^ However, the role of MYEOV in normal tissues, including the eye, has not been investigated to date. Our previous comparative RNA sequencing (RNA-seq) and epigenetic analyses revealed high MYEOV levels in terminally differentiated corneal epithelial cells and showed that MYEOV expression was epigenetically regulated by ten-eleven translocation 2 (TET2).^20^

In the current study, we aimed to further characterize the expression and regulation of MYEOV in the human ocular surface epithelium. We demonstrated that, among the surface ectoderm-derived tissues, MYEOV is highly expressed in KRT12-positive corneal epithelium and that its expression is regulated by PAX6 and KLF4.

## Materials and Methods

### Human Cell Source

The human tissue experiments complied with the guidelines of the ARVO Best Practices for Using Human Eye Tissue in Research. Human whole eye globes for immunostaining and corneas for cell culture studies were obtained from consented donors from the US eye banks Saving Sight (Kansas City, MO, USA) and CorneaGen (Seattle, WA, USA). Donor characteristics are described in Supplementary Table S1. Corneal epithelial cells for cell culture were harvested from the donor corneas as reported previously.^21^^,^^22^ Briefly, the central corneas were punched out by using an 8-mm disposable biopsy punch (Integra LifeSciences, Princeton, NJ, USA), and the corneal endothelium was mechanically removed. After a 1-hour incubation with PluriSTEM Dispase II Solution (MilliporeSigma, Burlington, MA, USA) at 37°C, corneal epithelial cells were scraped and dissociated by TrypLE Express Enzyme (Thermo Fisher Scientific, Waltham, MA, USA) at 37°C for 30 minutes. The cells were cultured in Dulbecco's Modified Eagle Medium/Nutrient Mixture F-12 (DMEM/F-12; Thermo Fisher Scientific) supplemented with 10 ng/mL keratinocyte growth factor (KGF; PeproTech, Cranbury, NJ, USA), 10 µM Y-27632 (Tocris Bioscience, Bristol, UK), and B-27 Supplement (Thermo Fisher Scientific).^23^ The cells were used within the three passages.

### Rodent Cell Source

The 3T3-J2 cell line (Kerafast, Boston, MA, USA) was maintained in DMEM (Thermo Fisher Scientific) supplemented with 10% calf serum (GE Healthcare Life Sciences, Chicago, IL, USA). 3T3-J2 cells were used as feeder cells for the colony-forming assay.

### Immunofluorescence Staining

Whole eye globes were immersed in 10% neutral buffered formalin and kept at 4°C overnight. The fixed eyes were then transferred to 70% ethanol. The specimens were embedded in paraffin at the BWH Pathology Core. Tissues were sectioned into 5-µm sections using a microtome. Deparaffinization and antigen retrieval were performed prior to the antibody staining steps. Permeabilization and blocking were performed using a buffer solution containing 5% normal donkey serum (Jackson ImmunoResearch Laboratories, West Grove, PA, USA) and 0.3% Triton X-100 (MilliporeSigma) for 30 minutes at room temperature. The sections were incubated with primary antibodies overnight at 4°C. The primary antibodies used were rabbit anti-MYEOV pAb (1:100, HPA012949; Atlas Antibodies, Stockholm, Sweden), mouse anti-KRT12 mAb (1:100, sc-515882; Santa Cruz Biotechnology, Santa Cruz, CA, USA), goat anti-KRT13 pAb (1:200, PA5-19049; Thermo Fisher Scientific), mouse anti-KRT10 mAb (1:100, sc-23877; Santa Cruz Biotechnology), and mouse anti-KRT14 pAb (1:100, sc-53253; Santa Cruz Biotechnology). The sections were rinsed with Tris-buffered saline (TBS; Boston BioProducts, Milford, MA, USA) and then incubated with Alexa Fluor 488–conjugated mouse secondary antibody (Abcam, Cambridge, UK), Alexa Fluor 568–conjugated rabbit secondary antibody (Abcam), and Alexa Fluor 647–conjugated goat secondary antibody (Abcam) for 1 hour at room temperature and stained with Hoechst 33342 (Thermo Fisher Scientific) for 10 minutes at room temperature. After being rinsed with TBS, the sections were mounted using ProLong Gold Antifade Mountant (Thermo Fisher Scientific). The samples were then imaged using a C2+ confocal microscope (Nikon, Tokyo, Japan), and captured images were analyzed by NIS-Elements AR 4.30.01 (Nikon).

### Re-Analyses of Single-Cell RNA Sequencing

The raw 10x single-cell sequencing data were downloaded from the National Center for Biotechnology Information Gene Expression Omnibus (GEO) database (GEO number GSE155683).^24^ The Uniform Manifold Approximation and Projection (UMAP) dimensionality reduction coordinates and cell type annotations were downloaded from the UCSC Cell Browser. The Seurat 4.2.0 package in R 4.1.2 (R Foundation for Statistical Computing, Vienna, Austria) was used to integrate the raw data, UMAP coordinates, and cell type annotations.^25^ Cells with unique molecular identifier counts less than 200 and without cell type annotations were filtered out. The gene expression counts were normalized to natural log-transformed expression values using the NormalizeData() function with the LogNormalize option in Seurat. Then, the VlnPlot function from the Seurat R package was used to generate violin plots for each cell type.

### RNA Interference

RNA interference was conducted by transfecting Silencer Select small interfering RNAs (siRNAs; Thermo Fisher Scientific) with Lipofectamine RNAiMAX Transfection Reagent (Thermo Fisher Scientific) following an established protocol.^26^ The siRNAs used were *Silencer* Select Negative Control No.1 siRNA, *PAX6* siRNAs (KD#1: s529237, KD#2: s529238, KD#3: s529239, KD#4: s10067, KD#5: s10068), *KLF4* siRNAs (KD#1: s17793, KD#2: s17794, KD#3: s17795), and *MYEOV* siRNAs (KD#1: s25554, KD#2: s25556).

### Reverse Transcription and Quantitative PCR

Total RNA was extracted from cultured corneal epithelial cells using the RNeasy Plus Mini Kit (QIAGEN, Hilden, Germany) according to the manufacturer's instructions. The extracted total RNA was converted into cDNA using the High-Capacity cDNA Reverse Transcription Kit (Thermo Fisher Scientific). Quantitative PCR (qPCR) was then performed with the TaqMan Fast Universal PCR Master Mix (Thermo Fisher Scientific) and the following TaqMan Gene Expression Assay probes: *GAPDH* (Hs99999905_m1), *MYEOV* (Hs00371084_m1), *KRT12* (Hs00165015_m1), *PAX6* (Hs01088114_m1), and *KLF4* (Hs00358836_m1) (Thermo Fisher Scientific). The qPCR reactions were run on a StepOnePlus Real-Time PCR System (Thermo Fisher Scientific). The cycling condition was 95°C for 20 seconds and then 50 cycles of 95°C for 1 second and 60°C for 20 seconds. Relative gene expression levels were calculated by normalizing to *GAPDH*.

### Western Blot Analyses

Cultured corneal epithelial cells underwent lysis using radioimmunoprecipitation assay (RIPA) buffer (Cell Signaling Technology, Danvers, MA, USA) supplemented with cOmplete Protease Inhibitor Cocktail (MilliporeSigma). The cell lysates were kept on ice for 30 minutes and then centrifuged to remove the cellular debris. Protein concentration was determined by Bio-Rad Protein Assay (Bio-Rad Laboratories, Hercules, CA, USA). After being mixed with SDS Sample Buffer (Boston BioProducts) and 2-mercaptoethanol (MilliporeSigma), the lysates were heated at 95°C for 10 minutes. The denatured proteins were separated using sodium dodecyl sulfate–polyacrylamide gel electrophoresis (SDS-PAGE) and subsequently transferred onto polyvinylidene fluoride blotting membranes (GE Healthcare Life Sciences). The membranes were blocked for 1 hour at room temperature using a buffer containing 5% blotting-grade blocker (Bio-Rad Laboratories) and then incubated with primary antibodies overnight at 4°C. Primary antibodies used were rabbit anti-β-actin pAb (1:1000, 5125; Cell Signaling Technology), rabbit anti-MYEOV pAb (1:1000, MBS9611913; MyBioSource, San Diego, CA, USA), rabbit anti-KRT12 mAb (1:5000, ab185627; Abcam), rabbit anti-PAX6 mAb (1:1000, ab195045; Abcam), and mouse anti-KLF4 mAb (1:500, sc-166238; Santa Cruz Biotechnology). After being rinsed with Tris-buffered saline with Tween 20 (TBST; MilliporeSigma), the membranes underwent a 1-hour incubation at room temperature with horseradish peroxidase (HRP)-conjugated mouse or rabbit secondary antibody (Cell Signaling Technology). The protein signals were visualized using Western Lightning Plus-ECL (PerkinElmer, Waltham, MA, USA), and images were captured with a ChemiDoc MP Imaging System (Bio-Rad Laboratories).

### Colony-Forming Assay

A colony-forming assay was conducted using a previously reported protocol.^21^^,^^22^ Briefly, trypsinized corneal epithelial cells were seeded on the 3T3-J2 feeder cell layer treated with mitomycin C (MMC; MilliporeSigma) at a density of 500 cells per well in six-well plates and were maintained for 10 days in keratinocyte culture medium (KCM) supplemented with 10 ng/mL KGF and 10 µM Y-27632. KCM is a complex mixture consisting of DMEM without glutamine and Ham's F-12 Nutrient Mix (Thermo Fisher Scientific) in a 3:1 ratio, supplemented with 10% FBS, 0.4 µg/mL hydrocortisone hydrogen succinate (MilliporeSigma), 2-nM 3,3′,5-triiodo-l-thyronine sodium salt (MilliporeSigma), 1-nM cholera toxin (List Biological Laboratories, Campbell, CA, USA), 2.25 µg/mL bovine transferrin HOLO form (Thermo Fisher Scientific), 2-mM l-glutamine (Thermo Fisher Scientific), 0.5% (vol/vol) insulin transferrin selenium solution (Thermo Fisher Scientific), and 1% (vol/vol) penicillin–streptomycin solution (GE Healthcare Life Sciences).^27^ The resulting colonies were fixed using 10% neutral buffered formalin and visualized by staining with rhodamine B (MilliporeSigma). The colony-forming efficiency was determined by calculating the ratio of the number of colonies formed to the initial number of cells seeded (500).

### Cell Proliferation Assay

The cell proliferation assay was performed using the Click-iT Plus EdU Flow Cytometry Assay Kit (Thermo Fisher Scientific) according to the manufacturer's protocol. The 5-ethynyl-2′-deoxyuridine (EdU) was added to the culture medium (final concentration was 20 µmol/L) and incubated for 12 hours before the cells were harvested. The EdU signal was detected by the FACSymphony A3 (BD Biosciences, Franklin Lakes, NJ, USA).

### RNA Sequencing

Genomic DNA contamination was eliminated from RNA samples using the DNA-free DNA Removal Kit (Thermo Fisher Scientific). The RNA was then submitted to Zymo Research (Irvine, CA, USA) to perform RNA-seq. After the removal of rRNA,^28^ approximately 500 ng of total RNA underwent library preparation with the Zymo-Seq RiboFree Total RNA Library Prep Kit (Zymo Research). Sequencing was carried out on an Illumina NovaSeq to a sequencing depth of 30 million read pairs (150 bp paired-end sequencing) per sample. Adaptor and low-quality sequences were trimmed from the raw reads, and the trimmed reads were aligned to the reference genome using the STAR 2.6.1d program.^29^ Differential expression analyses were performed by the DESeq2 1.28.0 R package.^30^

### Statistical Analysis

The data are shown as mean ± SD, and Dunnett's tests were performed to compare the siRNA-treated samples with negative control samples. *P < 0.05, **P < 0.01, ***P < 0.001, ****P < 0.0001.

### Data Availability

The RNA-seq data were deposited in the GEO database under accession number GSE295973.

## Results

### MYEOV Is Highly Expressed in KRT12-Positive Corneal Epithelial Cells

**Figure 1.**
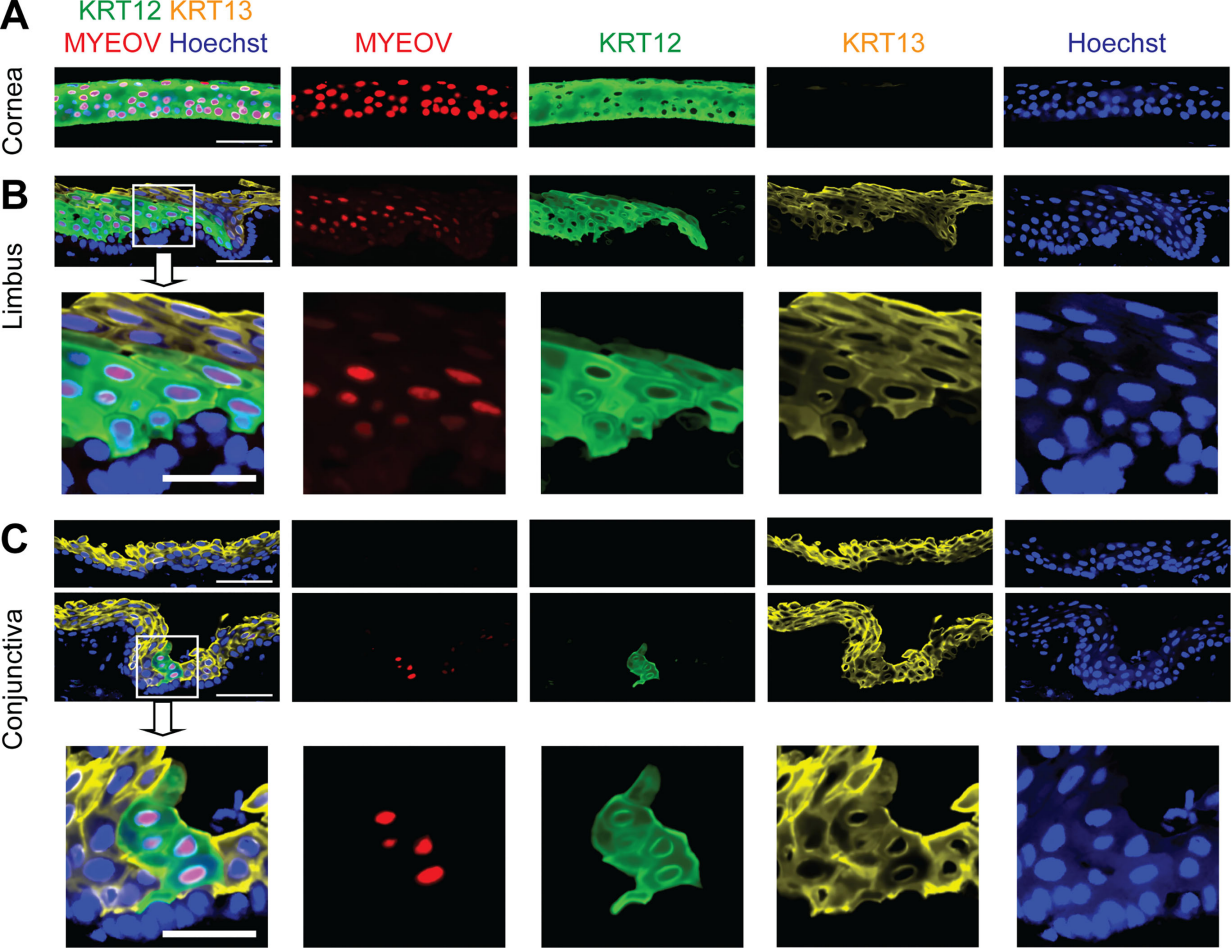
Expression of MYEOV in the human ocular surface. (**A**–**C**) Representative immunostaining analyses of MYEOV (*red*), KRT12 (*green*), and KRT13 (*yellow*) expression in the human cornea (**A**), limbus (**B**), and conjunctiva (**C**). Nuclei were visualized with Hoechst 33342 (*blue*). *White squares* depict the areas shown at the higher magnification in the panel below. *N* = 3 donors. *Scale bar*: 50 µm (25 µm for the magnified images).

We examined MYEOV expression in the human ocular surface. MYEOV was strongly expressed in the nuclei of KRT12-positive differentiated corneal epithelial cells in the cornea (Fig. 1A) and limbus (Fig. 1B). In contrast, the majority of the KRT13-positive differentiated conjunctival epithelial cells were MYEOV negative (Fig. 1C). Strong nuclear MYEOV expression was also observed in ectopic KRT12-positive cells in the conjunctiva (Fig. 1C).

**Figure 2.**
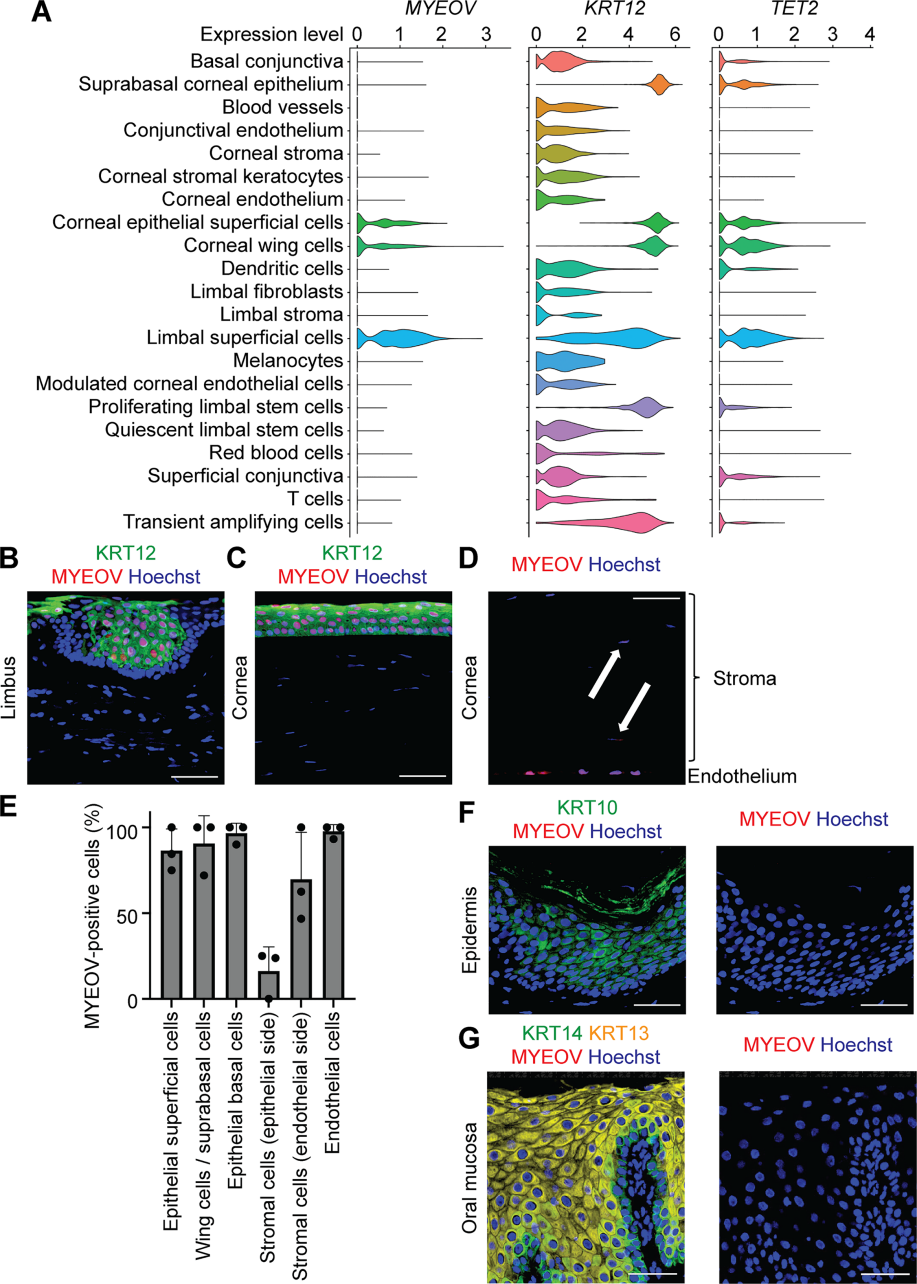
MYEOV expression in the eye, skin, and oral mucosa. (**A**) Violin plots depict the natural log-based normalized expression values of *MYEOV*, *KRT12*, and *TET2* in the human ocular surface. Plots were generated from GSE155683.^24^ (**B**, **C**) Representative immunostaining analyses of KRT12 (*green*) and MYEOV (*red*) expression in human corneal epithelium and stroma in the limbus (**B**) and cornea (**C**). (**D**) Representative immunostaining analyses of MYEOV (*red*) expression in human corneal endothelium and stroma. (**E**) The bar graph represents the percentage of MYEOV-positive cells in the human cornea detected by immunostaining. *Error bars* represent SD. (**F**) Representative immunostaining analyses of KRT10 (*green*) and MYEOV (*red*) expression in human epidermis. (**G**) Representative immunostaining analyses of KRT14 (*green*), KRT13 (*yellow*), and MYEOV (*red*) expression in human oral mucosa. Nuclei were visualized with Hoechst 33342 (*blue*). *N* = 3 donors. *Scale bar*: 50 µm.

Consistent with these observations, re-analyses of publicly available single-cell RNA-seq (scRNA-seq) data for human ocular surface tissue (GSE155683)^24^ revealed that *MYEOV* is mainly expressed in the differentiated corneal/limbal epithelial cells (corneal epithelial superficial cells, corneal wing cells, and limbal superficial cells) (Fig. 2A). This pattern is similar to that of *KRT12*. Still, *MYEOV* appears more specific to the differentiated corneal epithelial cells than *KRT12*. Despite the low *MYEOV* RNA level, some corneal stromal cells, mainly on the corneal endothelial side, were weakly positive for MYEOV protein staining (Figs. 2B–D). Furthermore, the majority of the corneal endothelial cells were positive for MYEOV protein expression despite the low *MYEOV* RNA level (Fig. 2D). The percentages of MYEOV-positive cells observed by immunostaining were 86.5% ± 12.6% in epithelial superficial cells, 90.7% ± 16.2% in wing cells and suprabasal cells, 96.7% ± 5.8% in epithelial basal cells, 16.3% ± 14.1% in epithelial side stromal cells, 69.7% ± 27.4% in endothelial side stromal cells, and 97.8% ± 3.9% in endothelial cells (Fig. 2E). The scRNA-seq studies also confirmed *MYEOV* and *TET2* co-expression (Fig. 2A), which is consistent with our previous report.^20^ No detectable MYEOV expression was observed in the other surface ectoderm-derived epithelial tissues, such as the KRT10-positive epidermis (Fig. 2F) and KRT13-positive oral mucosa (Fig. 2G). KRT14 was used to mark the basal epithelial cells of the oral mucosa (Fig. 2G).

### MYEOV Expression Is Regulated by Transcription Factors PAX6 and KLF4

**Figure 3.**
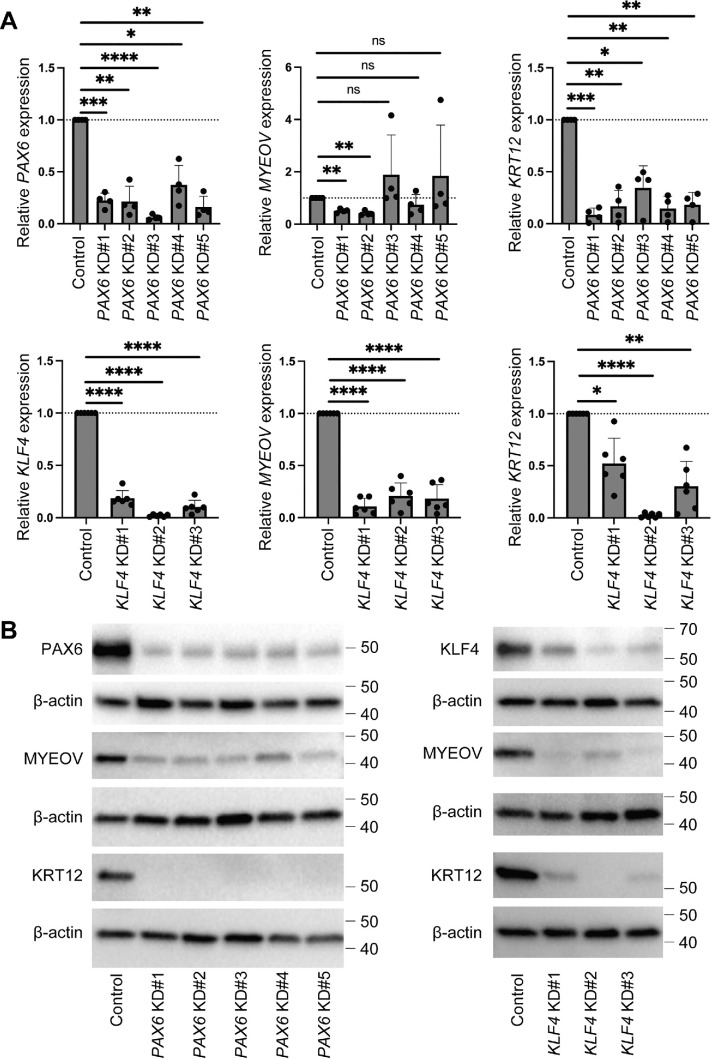
Regulation of MYEOV expression by PAX6 and KLF4 transcription factors. (**A**) Comparative analyses of *PAX6*, *KLF4*, *MYEOV*, and *KRT12* mRNA expression in the setting of *PAX6* KD using five distinct siRNAs and *KLF4* KD using three distinct siRNAs in cultured human corneal epithelial cells (*n* = 4 donors for *PAX6* KD, *n* = 6 donors for *KLF4* KD). *Error bars* represent SD. *P < 0.05, **P < 0.01, ***P < 0.001, ****P < 0.0001. (**B**) Representative western blot analysis of PAX6, KLF4, MYEOV, KRT12, and β-actin (loading control) protein expression by *PAX6* KD (*n* = 3 donors) and *KLF4* KD (*n* = 5 donors).

**Figure 4.**
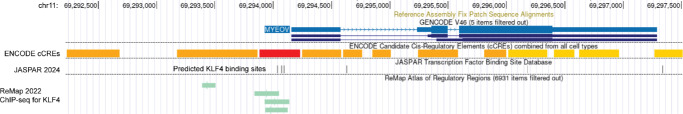
KLF4 binding sites within promoter/enhancer regions of *MYEOV*. KLF4 binding sites were mapped using data from JASPAR 2024^36^ and ReMap 2022 Atlas^37^ using the UCSC genome browser.^49^ ENCODE candidate *cis*-regulatory elements (cCREs) show the regions of promoter-like signature (*red*), proximal enhancer-like signature (*orange*), and distal enhancer-like signature (*yellow*) of *MYEOV.*^38^

Because MYEOV exhibits an expression pattern similar to that of KRT12, we next investigated the regulation of MYEOV by transcription factors PAX6 and KLF4, both of which are known to play critical roles in the induction of KRT12 expression.^6^^,^^26^^,^^31^^–^^35^ *PAX6* knockdown (KD) in cultured human corneal epithelial cells resulted in downregulation of KRT12 in both RNA and protein levels, but *MYEOV* RNA was not consistently regulated by PAX6 (Figs. 3A, 3B). However, *PAX6* KD led to the downregulation of MYEOV at the protein level, indicating an indirect effect of PAX6 in the regulation of MYEOV protein. On the other hand, *KLF4* KD resulted in the downregulation of KRT12 and MYEOV at both the RNA and protein levels (Figs. 3A, 3B). JASPAR 2024^36^ and ReMap 2022 Atlas^37^ showed multiple KLF4 binding sites within the regions of promoter-like signature (red) and proximal enhancer-like signature (orange) of *MYEOV* (Fig. 4).^38^ No PAX6 binding sites were detected within the regions of the promoter-like signature and proximal enhancer-like signature of *MYEOV*.

### *MYEOV* KD Reduced the Colony-Forming Efficiency

**Figure 5.**
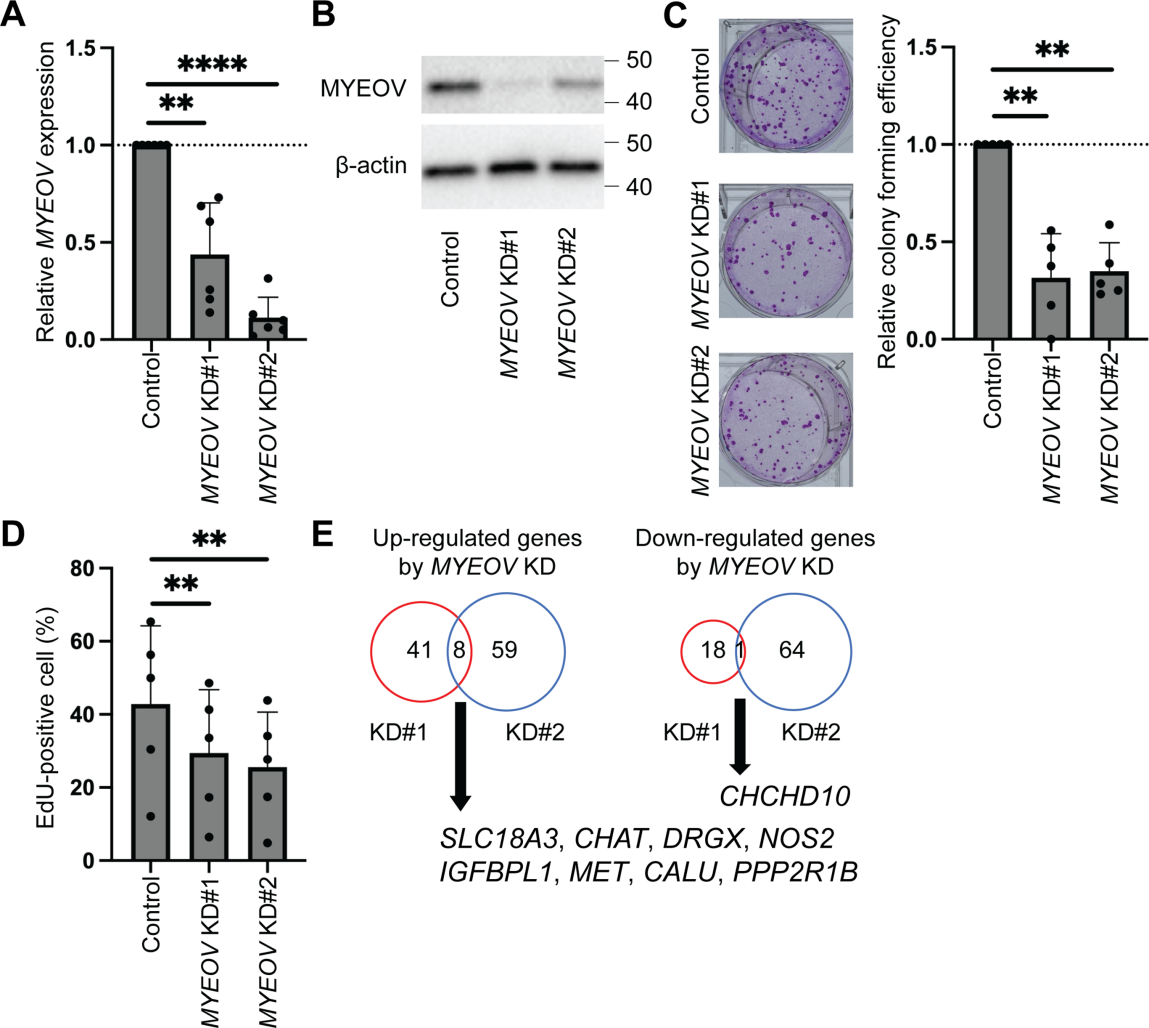
Functional role of MYEOV in corneal epithelial cells. (**A**) Comparative analyses of *MYEOV* mRNA expression by *MYEOV* KD using two distinct siRNAs in cultured human corneal epithelial cells (*n* = 6 donors). *Error bars* represent SD. **P < 0.01, ****P < 0.0001. (**B**) Representative western blot analysis of MYEOV and β-actin (loading control) protein expression in the setting of *MYEOV* KD (*n* = 4 donors). (**C**, *left panel*) Representative macroscopic images of the colonies formed by *MYEOV* KD cells compared to the control siRNA-transfected cells. (**C**, *right* *panel*) The bar graph represents comparative analyses of the colony-forming efficiency (*n* = 5 donors). *Error bars* represent SD. **P < 0.01. (**D**) Comparative analyses of the percentage of EdU-positive proliferating cells by *MYEOV* KD in cultured human corneal epithelial cells (*n* = 5 donors). *Error bars* represent SD. **P < 0.01. (**E**) Venn diagrams represent genes upregulated by *MYEOV* KD (*left*) and genes downregulated by *MYEOV* KD (*right*).

To reveal the function of MYEOV in corneal epithelium, *MYEOV* gene KD was performed using two distinct siRNAs (*MYEOV* KD#1 and *MYEOV* KD#2) and compared to a negative control siRNA. *MYEOV* KD was confirmed at both RNA (Fig. 5A) and protein levels (Fig. 5B). Cell proliferation was decreased, as evidenced by the reduced colony-forming efficiency (Fig. 5C) and EdU incorporation (Fig. 5D) in the setting of *MYEOV* KD. Interestingly, RNA-seq analyses revealed that *MYEOV* KD resulted in minimal changes in global gene expression. Only eight genes were upregulated (*SLC18A3*, *CHAT*, *DRGX*, *NOS2*, *IGFBPL1*, *MET*, *CALU*, and *PPP2R1B*), and one gene (*CHCHD10*) was downregulated by both *MYEOV* KD#1 and KD#2 (Fig. 5E, Supplementary Table S2).

## Discussion

In the current study, we investigated the expression and regulation of MYEOV in corneal epithelium. We found that MYEOV was highly expressed in KRT12-positive corneal epithelial cells throughout the ocular surface. We also found that most of the KRT13-positive conjunctival epithelial cells were MYEOV negative. Notably, MYEOV was not expressed in the other types of epithelia derived from surface ectoderm, such as epidermis and oral mucosa. Despite a low RNA level, some corneal stromal cells and most corneal endothelial cells expressed MYEOV protein.

Immunostaining showed very high expression of MYEOV in the whole layer of corneal epithelium, and these observations suggest that MYEOV has the potential to be used as a corneal epithelium-specific marker in addition to KRT12. Using both MYEOV and KRT12 as markers of the differentiated corneal epithelium could be helpful in multiple situations, such as research on corneal epithelial development, diagnosis of conjunctivalization of the ocular surface, and induction of corneal epithelial cells from embryonic stem cells^39^^–^^41^ or induced pluripotent stem cells.^41^^–^^43^ Additional research is required to investigate MYEOV expression patterns in the corneas of different species.

MYEOV expression was regulated by the TET2/5-hmC axis, as shown in our previous report.^20^ Re-analyses of scRNA-seq confirmed that *MYEOV* was highly expressed in *TET2*-positive cells.^24^ Besides TET2, the current study revealed regulation of MYEOV by transcription factors PAX6 and KLF4. PAX6 and KLF4 are known to regulate genes highly expressed in the corneal epithelium, such as *KRT12*, *ALDH3A1*, and *CLU.*^6^^,^^26^^,^^32^^–^^35^^,^^44^^,^^45^ This common regulatory mechanism between MYEOV and KRT12 seems to result in a similar pattern of their expression in the corneal epithelium. Although the RNA level of *MYEOV* was not consistently downregulated by all *PAX6* KD siRNAs, the protein level of MYEOV was downregulated by *PAX6* KD. MYEOV may be indirectly regulated by PAX6. For example, MYEOV protein could be stabilized by other proteins that are induced by PAX6. On the other hand, all *KLF4* KD conditions consistently downregulated the expression of MYEOV at both RNA and protein levels. JASPAR 2024 and ReMap 2022 Atlas data^36^^–^^38^ support the notion that KLF4 regulates the expression of *MYEOV* by directly binding to the promoter and proximal enhancer regions of *MYEOV*.

The colony-forming assay and EdU cell proliferation assay revealed a decreased cell proliferation by *MYEOV* KD in cultured corneal epithelial cells. Because cultured corneal epithelial cells are proliferative, they exhibit the characteristics of transient amplifying cells in the cornea. Together with the observation of MYEOV expression in the KRT12-positive basal epithelial cells in the cornea, these results suggest that MYEOV plays a role in maintaining proliferation in the transient amplifying cells in the cornea. The functional role of MYEOV in fully differentiated corneal epithelial cells requires further study.

RNA-seq data of *MYEOV* KD revealed that a minimal number of genes were affected by the expression levels of MYEOV. Among the upregulated genes in the setting of *MYEOV* KD, *IGFBPL1* and *PPP2R1B* are known to negatively regulate cell proliferation.^46^^,^^47^ In addition, *CHCHD10*, which was downregulated by *MYEOV* KD, is known to positively regulate cell proliferation.^48^ Therefore, MYEOV might promote transient amplifying cell proliferation through the negative regulation of *IGFBPL1* and *PPP2R1B* and the positive regulation of *CHCHD10*. The MYEOV–MYC association may regulate microRNA levels to promote cell proliferation, as reported in pancreatic ductal adenocarcinoma.^18^ Further studies are needed to reveal the underlying mechanisms. For upcoming studies, utilizing the CRISPR/Cas9 technique on human corneal epithelial cell lines to knockout *MYEOV* could be considered.

In conclusion, we have revealed specific high MYEOV expression in differentiated corneal epithelial cells among surface ectoderm-derived epithelia. Similar to KRT12, MYEOV expression is regulated by PAX6 and KLF4. Functionally, MYEOV contributes to the proliferation of transient amplifying cells in the cornea.

## Supplementary Material

Supplement 1
