# Supplementary material for: MYEOV Is a Novel Marker of Differentiated Corneal Epithelium
Source: Invest Ophthalmol Vis Sci. 2025 Nov 4;66(14):7. doi: 10.1167/iovs.66.14.7 (PMC12595593; doi:10.1167/iovs.66.14.7)
Supplement: Supplement 1 [file iovs-66-14-7_s001.docx]

**Supplementary Table 1**

Donor characteristics. N/A; not available.

| Age | Sex | Race | Death to preservation (hour:minutes) | Death to cell preparation (days) | Preservation | Cause of death | Figures |
| --- | --- | --- | --- | --- | --- | --- | --- |
| 58 | Male | Caucasian | 22:03 | 1 | Formalin | Probable hypertensive cardiovascular disease | 1A, 1B, 1C, 2B, 2C, 2D |
| 35 | Male | Caucasian | 06:35 | 1 | Formalin | Overdose | 1A, 1B, 1C |
| 61 | N/A | N/A | 05:43 | 1 | Formalin | COVID-19 | 1A, 1B, 1C, 2B, 2C, 2D |
| 73 | N/A | N/A | 17:15 | 1 | Formalin | Metastatic colon cancer | 2B, 2C, 2D |
| 33 | Female | Caucasian | 07:41 | 7 | Optisol-GS | Multi-system failure | 3A (*PAX6* KD) |
| 69 | Female | Caucasian | 09:14 | 7 | Optisol-GS | Cancer | 3A (*PAX6* KD), 3B (*PAX6* KD), 3B (*KLF4* KD) |
| 50 | Male | Caucasian | 08:59 | 8 | Optisol-GS | Myocardial infarction | 3A (*PAX6* KD), 3B (*PAX6* KD) |
| 65 | Male | Caucasian | 02:47 | 7 | Optisol-GS | Cancer | 3A (*PAX6* KD) |
| 64 | Female | Caucasian | 09:43 | 6 | Optisol-GS | Overdose | 3B (*PAX6* KD) |
| 59 | Female | Caucasian | 14:24 | 5 | Optisol-GS | Acute cardiac event | 3A (*KLF4* KD), 5D |
| 73 | Male | Caucasian | 17:45 | 5 | Optisol-GS | Acute cardiac event | 3A (*KLF4* KD) |
| 64 | Male | Caucasian | 12:29 | 9 | Optisol-GS | Acute cardiac event | 3A (*KLF4* KD), 3B (*KLF4* KD) |
| 72 | Male | Caucasian | 15:34 | 7 | Optisol-GS | Myocardial infarction | 3A (*KLF4* KD), 3B (*KLF4* KD) |
| 75 | Female | Caucasian | 07:18 | 3 | Moist chamber | Hemoperitoneum bleed | 3A (*KLF4* KD) |
| 72 | Male | Caucasian | 19:30 | 4 | Optisol-GS | Sudden Cardiac Event | 3A (*KLF4* KD), 3B (*KLF4* KD) |
| 75 | Male | Caucasian | 12:36 | 4 | Optisol-GS | Acute cardiac event | 3B (*KLF4* KD) |
| 78 | Male | Caucasian | 09:42 | 5 | Optisol-GS | Pulmonary fibrosis | 5A, 5B, 5E |
| 76 | Female | Caucasian | 06:08 | 4 | Optisol-GS | Acute cardiac event | 5A, 5B, 5E |
| 76 | Male | Caucasian | 13:44 | 4 | Optisol-GS | Acute cardiac event | 5A, 5E |
| 41 | Female | Caucasian | 14:44 | 5 | Optisol-GS | End-stage liver disease | 5A, 5B |
| 59 | Female | Asian | 08:52 | 4 | Optisol-GS | Cancer | 5A |
| 75 | Male | Caucasian | 10:47 | 3 | Optisol-GS | Chronic obstructive pulmonary disease | 5A, 5B |
| 63 | Male | Caucasian | 04:54 | 5 | Moist chamber | Glioblastoma multiforme | 5C |
| 73 | Male | Caucasian | 20:23 | 3 | Moist chamber | Cerebral vascular accident | 5C |
| 66 | Male | Caucasian | 08:14 | 4 | Moist chamber | Sepsis | 5C |
| 35 | Male | Caucasian | 05:13 | 6 | Optisol-GS | End-stage liver disease | 5C |
| 69 | Male | Caucasian | 06:13 | 5 | Moist chamber | Sepsis | 5C |
| 67 | Male | Caucasian | 18:40 | 4 | Moist chamber | N/A | 5D |
| 57 | Female | Black | 13:02 | 8 | Optisol-GS | Myocardial infarction | 5D |
| 74 | Female | Caucasian | 13:50 | 5 | Optisol-GS | Interstitial lung disease | 5D |
| 69 | Female | Caucasian | 25:32 | 8 | Kerasave | End-stage renal disease | 5D |

**Supplementary Table 2**

Up-regulated genes by *MYEOV* knockdown (KD)

| Gene Name | *MYEOV* KD #1 | | *MYEOV* KD#2 | |
| --- | --- | --- | --- | --- |
|  | log_2_ Fold Change | Adjusted p | log_2_ Fold Change | Adjusted p |
| *SLC18A3* | 6.35 | 0.010029 | 7.17 | 0.000478 |
| *CHAT* | 6.05 | 0.001607 | 5.70 | 0.002693 |
| *DRGX* | 5.80 | 0.027938 | 6.64 | 0.001587 |
| *NOS2* | 3.35 | 0.004623 | 2.99 | 0.018574 |
| *IGFBPL1* | 2.98 | 0.013162 | 3.22 | 0.001624 |
| *MET* | 1.79 | 0.000268 | 1.54 | 0.008782 |
| *CALU* | 1.62 | 0.001607 | 1.40 | 0.030644 |
| *PPP2R1B* | 1.51 | 0.000002 | 1.30 | 0.000524 |

A down-regulated gene by *MYEOV* knockdown (KD)

| Gene Name | *MYEOV* KD #1 | | *MYEOV* KD#2 | |
| --- | --- | --- | --- | --- |
|  | log_2_ Fold Change | Adjusted p | log_2_ Fold Change | Adjusted p |
| *CHCHD10* | -3.00 | 0.004569 | -2.56 | 0.022230 |
